# A Case of Septicemia due to Nonocclusive Mesenteric Ischemia Occurring in Induction Chemotherapy

**DOI:** 10.1155/2018/7426819

**Published:** 2018-04-19

**Authors:** Hideki Tanaka, Kiyoaki Tsukahara, Isaku Okamoto, Rio Kojima, Kazuhiro Hirasawa, Hiroki Sato

**Affiliations:** Department of Otorhinolaryngology, Head and Neck Surgery, Tokyo Medical University, Tokyo, Japan

## Abstract

In nonocclusive mesenteric ischemia (NOMI), mesenteric ischemia and intestinal necrosis occur despite the absence of organic blockage in mesenteric blood vessels. As abdominal pain is often absent and few characteristic findings are seen in blood biochemistry, imaging diagnosis or other examinations, discovery is often delayed. With a mortality rate of 56–79%, NOMI is a very serious disease. However, few reports have described this pathology in association with chemotherapy regimens such as those used for malignant head and neck tumors. We encountered a case of NOMI during induction therapy combining cisplatin, docetaxel, and 5-fluorouracil. The patient was a 74-year-old man receiving chemotherapy for T2N2bM0 stage IVA oropharyngeal carcinoma. Febrile neutropenia appeared on treatment day 8. An antibacterial agent and a granulocyte colony-stimulating factor were administered, but septic shock developed and he was transferred to the intensive care unit. Abdominal distension was present and contrast-enhanced computed tomography of the abdomen suggested NOMI. Emergency surgery on day 9 resected the necrotized small intestine and created a single-hole ileostomy. The patient subsequently recovered with 2 weeks of continuous hemodiafiltration and other intensive therapies. Otolaryngological surgeons seldom encounter intestinal diseases, which are thus easily overlooked. The present case report may help in achieving early diagnosis.

## 1. Introduction

Induction chemotherapy combining the 3 agents of cisplatin (CDDP), docetaxel (DOC), and 5-fluorouracil (5-FU) is standard treatment in head and neck cancer. This triple-therapy is also referred to as TPF (Taxotere, Platinol, and fluorouracil). As TPF is a powerful therapy, adverse events are frequent, and sometimes fatal.

Nonocclusive mesenteric ischemia (NOMI) is an intestinal blood flow disorder without blockage of the main trunk of the mesenteric artery. NOMI has few specific clinical symptoms and findings on medical examinations. As diagnosis is often delayed and irreversible intestinal necrosis results, NOMI is a serious disease. This pathology has a wide range of causes, and chemotherapy has been reported as one. However, no previous reports have described NOMI occurring as a complication of chemotherapy in head and neck cancer [1–4]. We report herein a case of NOMI due to induction chemotherapy with TPF for oropharyngeal carcinoma.

## 2. Case Details

**Figure 1:**
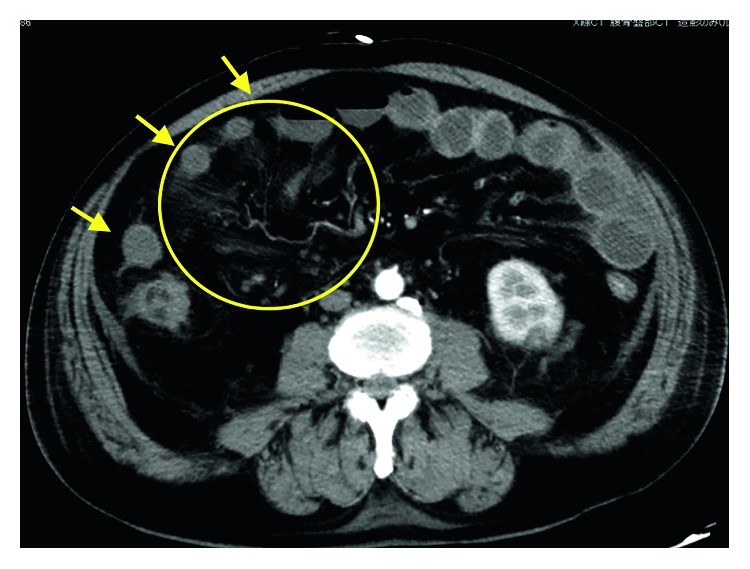
Axial-section contrast-enhanced CT of the abdomen performed in the ICU. Poor contrast effects can be seen in the small intestine (arrows). Disruption of peripheral blood vessels of the superior mesenteric artery is apparent (circled).

**Figure 2:**
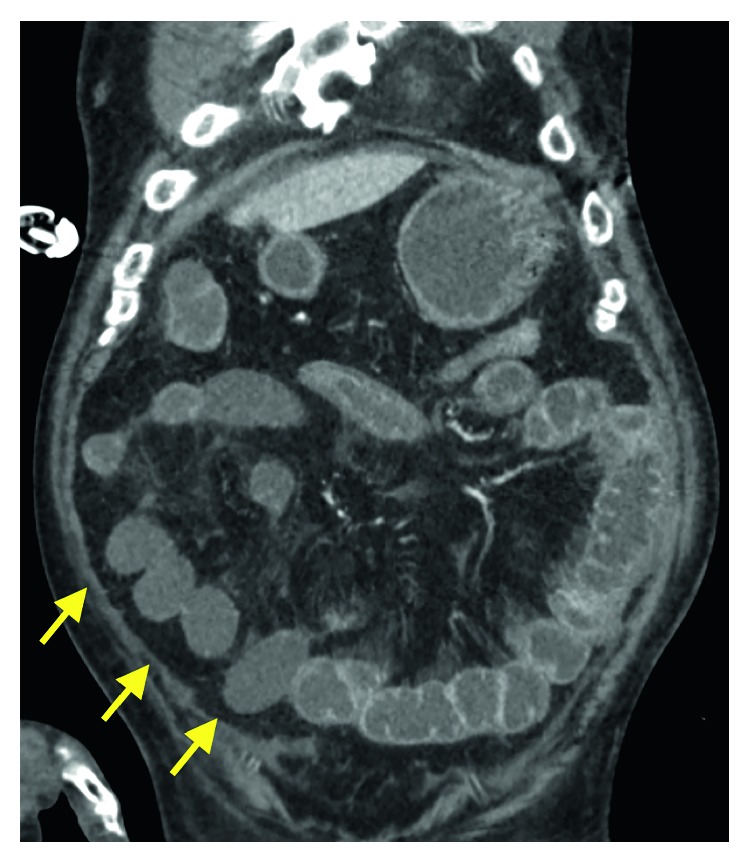
Coronal-section contrast-enhanced CT of the abdomen performed in the ICU. Poor contrast effects can be seen in the small intestine (arrows).

**Figure 3:**
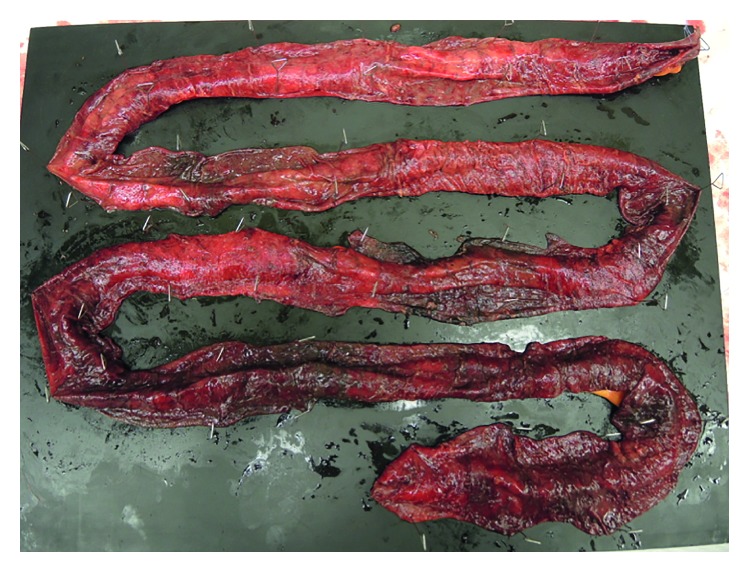
Resected small intestine. Multiple ischemic locations are seen, for around 200 cm from the ligament of Treitz to about 10 cm from the terminal ileum.

The patient was a 74-year-old man who was examined for the major complaint of swelling in the right neck. His medical history included pneumonia and sensorineural hearing loss, but no family history of note was elicited. He had smoked 40 cigarettes a day until 32 years old. He had first noted swelling of the right neck 5 years earlier, and had been referred to our department in April 2016 after noticing a growing tendency. Pharyngolaryngeal endoscopy had revealed swelling of the lateral wall on the right side of the oropharynx. On cervicothoracic contrast-enhanced computerized tomography (CT), a mass with a maximum diameter of 24 mm was noted in the lateral wall of the right oropharynx, but no infiltration into the parapharyngeal space or epiglottis was seen. Swelling was also observed in the right upper and middle deep neck lymph nodes and the right node of Rouviere. In blood biochemistry, a squamous cell carcinoma-related antigen was elevated to 2.8 ng/mL. Biopsy of the right oropharynx lateral wall revealed p40-and p16-positive squamous cell carcinoma. The diagnosis was T2N2bM0 stage IVA oropharyngeal carcinoma of the right lateral wall. The treatment plan was to manage the disease through induction chemotherapy with TPF. As induction chemotherapy, CDDP and DOC were both administered at 60 mg/m^2^ on day 1 and 5-FU at 600 mg/m^2^ from day 1 to day 5. Daily urine volume was ≥3,000 mL. Although bodyweight decreased day by day from day 4, compared to before the start of chemotherapy, no deterioration in patient condition had been seen; so, we kept the patient under observation with particular attention to vital signs. On day 8, neutrophil count had dropped to 2250/*μ*L and blood pressure fell to 70/50 mmHg. However, as no fever was evident, febrile neutropenia was ruled out. As fluid balance continued to be negative each day, we conducted fluid replacement for extracellular fluids to prevent dehydration. The diarrhea that appeared was considered to be drug-induced colitis due to 5-FU, and symptomatic treatment was initiated. On day 9, he showed a fever of 38.0°C, and neutrophil count had decreased to 212/*μ*L. Febrile neutropenia was diagnosed, and administration of cefepime (CFPM), an antibacterial agent, and a granulocyte colony-stimulating factor (G-CSF) agent was commenced. However, 3 h later, the patient lost consciousness and blood pressure fell to 50/40 mmHg. Septic shock was diagnosed, and he was transferred to the Intensive Care Unit (ICU). Treatment of the septic shock according to early goal-directed therapy was combined with continuous hemodiafiltration and polymyxin B-immobilized fiber column hemoperfusion. As nausea and marked abdominal distension were present, contrast-enhanced CT of the abdomen was conducted (Figures 1 and 2). While a decrease in contrast effect was observed in the small intestine wall, no prominent thrombosis of the mesenteric artery or veins was observed, so NOMI was considered. In consultation with our gastrointestinal surgery department, conservative therapy was considered problematic, and the decision was made to conduct emergency surgery. Partial resection of the small intestine was performed and an ileostomy was created. Numerous ischemic locations were seen in the small intestine, and about 1 m of the intestine was resected (Figure 3). No intestinal perforation or intraperitoneal contamination was observed. Postoperative pathological examination showed necrosis in the intestinal wall but no organic blockage or blood clots in the mesenteric artery.

On day 2 after surgery, the shock abated. On day 12, the patient was returned to the general ward from the ICU. Contrast-enhanced CT of the neck on postoperative day 15 showed partial response of the oropharyngeal carcinoma. As additional chemotherapy was considered problematic, treatment with cetuximab was conducted in combination with radiotherapy. Both the primary cancer and neck lymph node lesions subsequently disappeared. After half a year, the ileostomy was closed off. In the 9 months following the completion of treatment, no recurrence or metastasis was observed.

## 3. Discussion

NOMI is a disease of the mesenteric blood vessels without organic blockage that causes mesenteric ischemia and intestinal necrosis. This pathology is the cause of 15–27% of intestinal ischemia and shows a high mortality rate of 56–79% [5, 6]. However, patients frequently show few findings from blood biochemistry or imaging examinations. One reason for the high mortality rate is delayed discovery because of the lack of characteristic findings. Abdominal distension and abdominal pain were observed in the present case, but abdominal pain is not seen in 20–30% of cases [7–9].

The major features in the pathology of NOMI are an extreme drop and unevenness in intestinal blood flow. Another cause is peripheral vascular spasm due to exaggerated responses in sympathetic nerves and fluid factors, such as vasopressin and angiotensin, accompanying decreases in cardiac output and circulating plasma volume [10]. The three major risks are thus congestive heart failure, digitalis intoxication, and hemagglutination. Other causes include sympathomimetic drugs, diuretics, dialysis, and septic shock. However, chemotherapy has not been reported as a risk factor for NOMI.

A search of PubMed for NOMI and chemotherapy found only 4 cases of NOMI due to chemotherapy (Table 1). However, the sites of the primary cancer and regimens varied. Various adverse events occur with chemotherapy and may cause NOMI as a secondary effect. In the present case, daily decreases in bodyweight were seen from day 4 of administering TPF, and hemoconcentration, one of the risk factors for NOMI, occurred. Also, as septic shock resulted from febrile neutropenia, impaired blood flow in the intestinal mucosa was considered to have been exacerbated.

Drug-induced colitis may occur as an adverse event of the 5-FU used in TPF therapy. Symptoms are diarrhea and nausea in mild cases and hemorrhagic colitis and necrotizing enterocolitis in serious cases, resembling NOMI. However, major differences exist between these pathologies regarding treatment and prognosis. In 5-FU-induced colitis, 1.8% of all diarrhea that occurs may be fatal [11]. Furthermore, NOMI may be hidden among patients diagnosed with 5-FU-induced colitis.

With the NOMI diagnosis algorithm used in the United States, selective angiography has been suggested as the number-one diagnostic modality. However, when NOMI is discovered, systemic conditions are frequently unstable. The rate of conducting selective angiography is thus reportedly low [12]. Diagnostic criteria for mesenteric vascular spasm are proximal stenosis of the superior mesenteric artery and main branch, abnormal images of mesenteric branches, arcade spasm of the mesenteric artery, and poor contrast on internal images of the blood vessels in the mesenteric wall.

**Figure 4:**
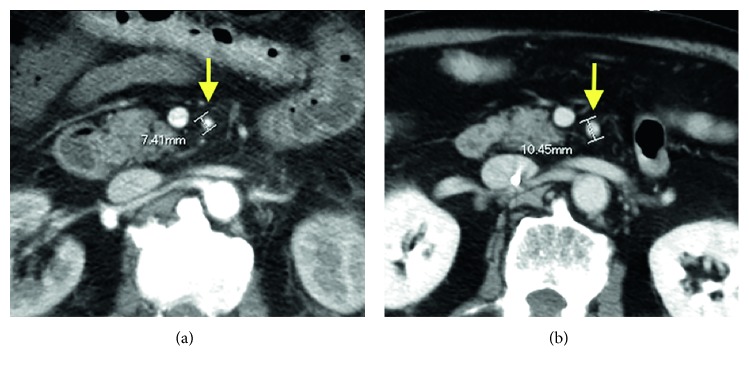
Coronal-section contrast-enhanced CT of the abdomen. (a) Before surgery. (b) 3 months after surgery. The diameter of the superior mesenteric artery is 7.41 mm before surgery and 10.45 mm at 3 months after surgery (arrows).

The appearance of multidetector row CT (MDCT) in recent years has improved the visualization of veins. MDCT allows angiography diagnostic criteria-related findings to be evaluated [13]. With MDCT, it is also possible to diagnose loss of blood flow contrast effect in the intestinal wall and thinning of the intestinal wall due to decreased mesenteric blood flow. Intestinal ischemia is regional in nature, from the small intestine to the colon, and is distributed unevenly. Regarding the present case, the presence of an extensive poor contrast effect in the intestinal wall and vascular disruption in the superior mesenteric artery periphery on MDCT led to the diagnosis of NOMI. Compared with the results of CT 3 months after surgery, the superior mesenteric artery appeared narrowed on CT before surgery (Figure 4). However, angiography should be performed in accordance with the American standards where possible. Regrettably, due to the emergency nature of the intervention and in-hospital circumstances, we were unable to perform angiography.

Thinking that the present case represented 5-FU-induced colitis, the patient had initially been put under observation, with only fluid replenishment and symptomatic treatment. If we had performed MDCT at that time, the disease state may not have become as serious. The drug 5-FU and its derivatives are a major part of treatment in head and neck cancer, and inclusion of NOMI among the differential diagnoses of colitis is therefore important.

Treatment of NOMI can be divided into surgical and nonsurgical methods. For the latter, continuous intra-arterial infusion of vasodilators, such as papaverine hydrochloride, is useful. Peritoneal irritation symptoms are often an indicator for surgery. However, in many cases, physical findings cannot be relied upon in elderly patients and those in a poor state of consciousness. Nakao et al. actively performed surgery without angiography for 17 patients with NOMI complicated by shock, and found that colectomy was necessary in 14 patients (82%) [14].

If an irreversible ischemic state is determined to have developed in the large intestine, as in the present case, surgery is absolutely necessary. In our patient, prompt diagnosis and surgical intervention enabled early infection control, and the early discovery of NOMI potentially saved his life. The emphasis should thus be on early diagnosis of NOMI by means of contrast-enhanced MDCT and teamwork in therapy involving intensive care specialists and gastroenterological surgeons.

## 4. Conclusion

We encountered a rare case of NOMI occurring during induction chemotherapy for head and neck cancer. Otolaryngology department surgeons such as ourselves seldom encounter intestinal diseases and may overlook them. For NOMI in particular, characteristic clinical findings are few, and differentiation from 5-FU-induced colitis is difficult. However, contrast-enhanced MDCT is useful for distinguishing between these pathologies. The unstable hemodynamics in chemotherapy may cause NOMI. The association between chemotherapy and NOMI should be verified in a larger population.

## Figures and Tables

**Table 1:** Previously reported case of NOMI during chemotherapy.

Author	Age/sex	Cancer region	Chemotherapy regimen	Year reported
Pearson et al. [3]	53/female	Metastatic liver cancer	CDDP, ADM, MMC	2008
Awano et al. [1]	80/female	Lung Adenocarcinoma	gefitinib	2013
Matsuzawa et al. [4]	74/female	Melanoma	DTIC, ACNU, VCR, ß-interferon	2015
Yamane et al. [2]	68/male	Small cell lung cancer	CDDP, ETP	2015
Present case	74/male	Oropharynx cancer	CDDP, 5-FU, DOC	2017

CDDP: cisplatin; ADM: adriamycin; MMC: mitomycin C; CBDCA: carboplatin; DTIC: dacarbazine; ACNU: nimustine; VCR: vincristine; ETP: etoposide; DOC: docetaxel.
